# Clinical features and therapeutic outcomes of GH/TSH cosecreting pituitary adenomas: experience of a single pituitary center

**DOI:** 10.3389/fendo.2023.1197244

**Published:** 2023-05-30

**Authors:** Na Yu, Lian Duan, Fang Hu, Shengmin Yang, Jie Liu, Meiping Chen, Yong Yao, Kan Deng, Feng Feng, Xin Lian, Xinxin Mao, Huijuan Zhu

**Affiliations:** ^1^ Key Laboratory of Endocrinology of National Health Commission, Department of Endocrinology, State Key Laboratory of Complex Severe and Rare Diseases Peking Union Medical College Hospital, Chinese Academy of Medical Science and Peking Union Medical College, Beijing, China; ^2^ Department of Endocrinology and Metabolism, The Fifth Affiliated Hospital, Sun Yat-Sen University, Zhuhai, Guangdong, China; ^3^ Department of Neurosurgery, Peking Union Medical College Hospital, Chinese Academy of Medical Science and Peking Union Medical College, Beijing, China; ^4^ Department of Radiation Oncology, Peking Union Medical College Hospital, Chinese Academy of Medical Sciences and Peking Union Medical College, Beijing, China; ^5^ Department of Pathology, Peking Union Medical College Hospital, Chinese Academy of Medical Science and Peking Union Medical College, Beijing, China

**Keywords:** GH/TSH cosecreting pituitary adenoma, clinical features, therapeutic outcomes, multidisciplinary therapy, pituitary center

## Abstract

**Background:**

Growth hormone (GH)/thyroid stimulating hormone (TSH) cosecreting pituitary adenoma (PA) is an exceedingly rare kind of bihormonal pituitary neuroendocrine tumors (PitNETs). Its clinical characteristics have rarely been reported.

**Objectives:**

This study aimed to summarize the clinical characteristics and experience of diagnosis and treatment among patients with mixed GH/TSH PAs from a single center.

**Methods:**

We retrospectively reviewed GH/TSH cosecreting PAs from 2063 patients diagnosed with GH-secreting PAs admitted to Peking Union Medical College Hospital between January 1^st^, 2010, and August 30^th^, 2022, to investigate the clinical characteristics, hormone detection, imaging findings, treatment patterns and outcomes of follow-up. We further compared these mixed adenomas with age- and sex-matched cases of GH mono-secreting PAs (GHPAs). The data of the included subjects were collected using electronic records from the hospital’s information system.

**Results:**

Based on the inclusion and exclusion criteria, 21 GH/TSH cosecreting PAs were included. The average age of symptom onset was 41.6 ± 14.9 years old, and delayed diagnosis occurred in 57.1% (12/21) of patients. Thyrotoxicosis was the most common complaint (10/21, 47.6%). The median inhibition rates of GH and TSH in octreotide suppression tests were 79.1% [68.8%, 82.0%] and 94.7% [88.2%, 97.0%], respectively. All these mixed PAs were macroadenomas, and 23.8% (5/21) of them were giant adenomas. Comprehensive treatment strategies comprised of two or more therapy methods were applied in 66.7% (14/21) of patients. Complete remission of both GH and TSH was accomplished in one-third of cases. In the comparison with the matched GHPA subjects, the mixed GH/TSH group presented with a higher maximum diameter of the tumor (24.0 [15.0, 36.0] mm *vs*. 14.7 [10.8, 23.0] mm, P = 0.005), a greater incidence of cavernous sinus invasion (57.1% *vs*. 23.8%, P = 0.009) and a greater difficulty of long-term remission (28.6% *vs*. 71.4%, P <0.001). In addition, higher occurrence rates of arrhythmia (28.6% *vs*. 2.4%, P = 0.004), heart enlargement (33.3% *vs*. 4.8%, P = 0.005) and osteopenia/osteoporosis (33.3% *vs*. 2.4%, P = 0.001) were observed in the mixed PA group.

**Conclusion:**

There are great challenges in the treatment and management of GH/TSH cosecreting PA. Early diagnosis, multidisciplinary therapy and careful follow-up are required to improve the prognosis of this bihormonal PA.

## 1 Introduction

Pituitary tumors account for nearly 15% of central nervous system tumors, most of which are pituitary adenomas (PAs), which have recently been renamed as pituitary neuroendocrine tumors (PitNETs) in the latest World Health Organization (WHO) classification (1, 2). According to whether hormones are overproduced, PitNETs are divided into clinically functional and nonfunctional PAs (NFPAs). Functional tumors cause conditions related to hormonal hypersecretion, such as elevated plasma growth hormone (GH) and/or insulin-like growth factor 1 (IGF-1), leading to acromegaly and inappropriate secretion of thyroid stimulating hormone (TSH), causing central hyperthyroidism (3). As a special type of PA, GH/TSH cosecreting PA is rarer and secretes GH and TSH simultaneously. GH-producing PAs accounts for 5.7%-16.5% of PAs, whereas TSH-producing PAs occupy 0.5%-3.0% of PAs (4–8). Furthermore, GH/TSH cosecreting PAs were much rarer, which take up only 16.0%-19.7% of TSH-producing PAs (9, 10). Due to the extraordinarily low occurrence of GH/TSH cosecreting PAs, most related papers are single-case reports, and the implications are limited (11–20). Since two hormones may have synergistic interactions in clinical manifestations, treatment responses and follow-up outcomes, the comparison of plurihormonal PA and monohormonal PA should be encouraged. A previous study by Li et al. has reported the clinical features of twelve mixed GH/TSH PAs which were compared with TSH monosecreting macroadenomas, demonstrating the former has a higher proportion of cavernous sinus invasion and a lower surgical complete remission rate (21). However, the comparative analysis between GH/TSH cosecreting PAs and GH monosecreting PAs has not been fully studied by far. Therefore, this study aimed to review cases of GH/TSH cosecreting PAs in Peking Union Medical College Hospital (PUMCH), one of the largest pituitary centers in China, for over 12 years and compare the rare mixed GH/TSH PAs with GH monosecreting PAs.

## 2 Methods

### 2.1 Diagnostic criteria and study population

The diagnostic criteria of GH-producing pituitary adenoma (GHPA) were as follows: (i) clinical signs and symptoms related to excessive GH and IGF-1; (ii) nadir GH exceeding 1.0 ng/ml with a 75 g oral glucose tolerance test (OGTT) and supranormal IGF-1 levels adjusted for age and sex; and (iii) pituitary adenoma by magnetic resonance imaging (MRI). Mixed GH/TSH PAs were additionally identified with TSHoma. The diagnosis of TSHoma was confirmed by the following points: (i) clinical symptoms of thyrotoxicosis (absent, mild, severe); (ii) high serum free thyroxine (FT4) and free triiodothyronine (FT3) with normal or elevated TSH concentrations; (iii) pituitary adenoma on MRI; and (iv) no mutations in the gene encoding thyroid hormone receptor β (*THRB*).

This is a retrospective study of 21 consecutive patients diagnosed with GH/TSH cosecreting PA who were admitted to PUMCH from January 1st, 2010 to August 30th, 2022. Only patients who met the following criteria were included: (i) sufficient medical records, especially the available materials before initial therapy; (ii) regular follow-up of no less than 6 months after treatment; and (iii) no history of surgery or radiotherapy targeting the pituitary gland. The exclusion criteria were as follows: (i) patients with other thyroid diseases. (ii) cosecretion of prolactin (or other anterior pituitary hormones) in PA. The control group included age-matched and sex-matched patients with GH monosecreting PA for comparison with the GH/TSH coexisting PA group. The matching ratio was 1:2 (GH/TSH cosecreting adenoma cases *vs*. GH monosecreting adenoma cases) between the two groups.

The protocol and the procedures of the study were approved by the Ethics Committee of Peking Union Medical College Hospital (No. I-23PJ665). The entire study was conducted in accordance with the Code of Ethics of the World Medical Association (Declaration of Helsinki) for experiments involving humans.

### 2.2 Clinical data collection and definition of remission or relapse

The clinical data were extracted from patients’ medical records in the hospital’s information system, including general information (age at diagnosis, sex, disease duration, BMI, blood pressure, heart rate, chief complaints, vital signs, comorbidities), laboratory examinations, imaging inspection (MRI, thyroid ultrasound, echocardiography), histopathological findings, treatment patterns and follow-up outcomes of patients.

Biochemical remission of the GH/IGF-1 axis was prescribed as nadir GH of OGTT (or fasting GH at the outpatient clinic) <1.0 ng/ml and lower IGF-1 than ULNs. Biological remission of the TSH axis in coexisting adenoma was defined as levels of FT3, FT4 and TSH lower than their respective ULNs simultaneously. The definition of relapse was higher hormone levels beyond ULNs and/or regrowth of neuroradiological lesions after complete resection.

Abnormal glucose metabolism in this study included diabetes, impaired fasting blood glucose (IFG) and impaired glucose tolerance (IGT). Combined with the WHO (1999) standard, the criteria for abnormal glucose metabolism could be summarized as fasting blood glucose (FBG) ≥6.1 mmol/l and/or two-hour postprandial blood glucose ≥7.8 mmol/l, as well as cases previously diagnosed with diabetes. According to Chinese guidelines for prevention and treatment of dyslipidemia in adults (2016 revision), hyperlipidemia referred to serum total cholesterol (TC) ≥5.2 mmol/l (200 mg/dl) and/or triglyceride (TG) ≥1.7 mmol/l (150 mg/dl) or history of diagnosed hyperlipidemia. Based on the 2018 European Society of Cardiology (ESC)/European Society of Hypertension (ESH) guidelines for management of blood pressure, hypertension was defined as systolic blood pressure (SBP) ≥140 mmHg and/or diastolic blood pressure (DBP) ≥90 mmHg of history of hypertension diagnosis.

### 2.3 Laboratory, imaging and clinicopathological examinations

The octreotide test (OCT) was performed (0.1 mg, subcutaneously, once; measuring TSH levels after 0, 2, 4, 6, 8, 24, 48 and 72 hours; determining GH levels after 0, 2, 4, 6 and 8 hours), which was considered as eligible suppression if the levels of GH and TSH decreased to less than 50% of their baseline level. GH and IGF-1 were measured by automatic chemiluminescence enzyme immunoassay (Immulite 2000^®^; Siemens Healthcare Diagnostics, USA). The IGF-1 to ULN ratio (IGF-1/ULN) was transformed from the absolute value using age- and sex-matched ULNs at our institute to make IGF-1 comparable between patients. Thyroid hormones, including FT3, FT4 and TSH, were tested by the direct chemiluminescence method (ADVIA Centaur, Siemens, USA). The reference intervals of the laboratory in PUMCH were as follows: FT3, 1.80-4.10 pg/ml; FT4, 0.81-1.89 ng/dl; TSH, 0.38-4.34 μIU/ml. Following an overnight fast for at least 8 hours, blood samples were collected from all patients in the early morning. Serum glucose, hemoglobin A1c (HbA1c) and other hormones related to anterior pituitary function were determined by conventional automated laboratory methods in the clinical laboratory of PUMCH.

Enhanced 3.0 T pituitary MRI was used to noninvasively assess the pituitary lesions, which were analyzed by the experienced neuroradiologists. The tumors were classified into 3 types: microadenomas (<10 mm), macroadenomas (≥10 mm) and giant adenomas (≥40 mm) according to their maximum diameter. Somatostatin receptor scintigraphy was also performed with 99mTc-labeled octreotide injected intravenously to verify the diagnosis. Immunohistochemical analysis of pituitary adenoma tissue was performed on 4-5 µm paraffin sections uniformly using an automated IHC system (Leica, BOND-MAX, Wetzlar, Germany). H&E and immunostaining were assessed by the neuropathologists with expertise in pituitary pathology who were blinded to the clinical information.

### 2.4 Statistical analysis

The continuous variables were expressed as the mean ± SD or median with interquartile ranges if necessary. Categorical variables are represented as counts and percentages. Student’s *t* test and Mann−Whitney U test were performed for the comparison of continuous variables between two groups. Categorical variables were compared by Fisher’s exact test. A two-tailed P <0.05 was considered statistically significant in all analyses. Propensity-score matching analysis was performed to screen age- and sex-matched patients with GH monosecreting pituitary adenoma as the control group. The statistical analyses were performed with the statistical software packages R (http://www.R-project.org, The R Foundation) and Free Statistics software versions 1.7. 1.

## 3 Results

### 3.1 Study participants and baseline characteristics

**Figure 1:**
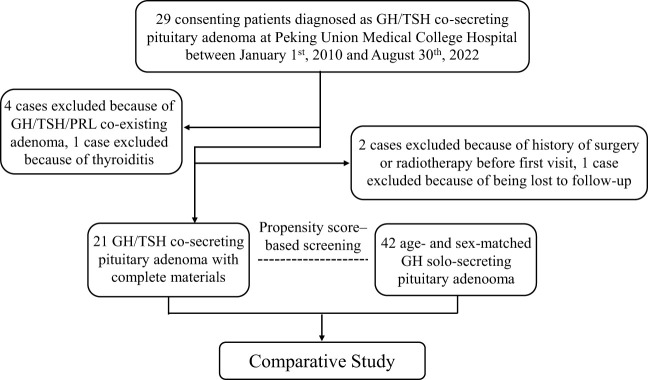
Flowchart of TSH/GH cosecreting pituitary adenoma inclusion and GH monosecreting pituitary adenoma screening as controls. GH, growth hormone; TSH, thyroid stimulating hormone; PRL, prolactin.

From January 1st, 2010 to August 30th, 2022, 2063 patients admitted to PUMCH for GH-secreting pituitary tumors and 29 patients were identified with GH/TSH cosecreting PA, accounting for 1.40% of the GH-secreting PAs. According to the inclusion and exclusion criteria, 21 patients with GH/TSH cosecreting pituitary adenoma were included in the final analysis (**Figure 1**). For the comparative study, 42 age- and sex-matched patients with GH monosecreting tumors were included as controls.

**Figure 2:**
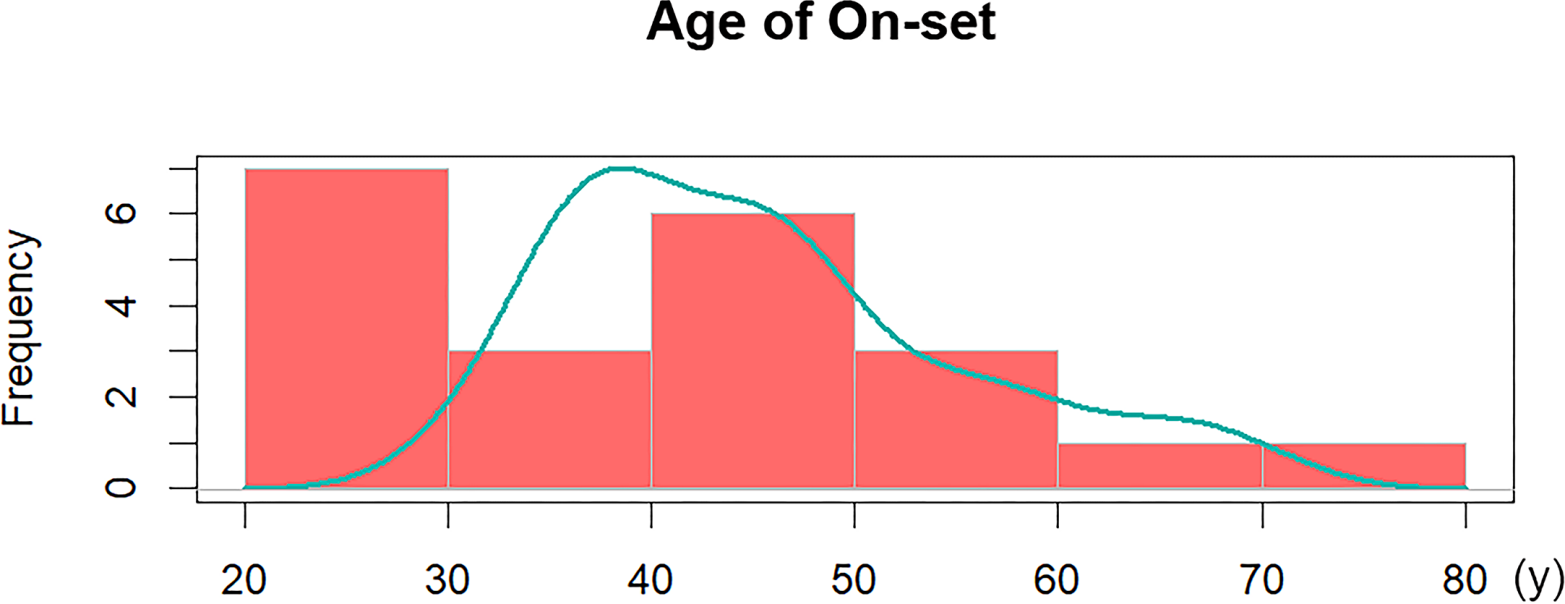
Age distribution of 21 GH/TSH cosecreting pituitary adenomas.

The baseline data of participants with mixed GH/TSH tumors, including age of admission or symptom onset, sex, delayed diagnosis and anthropometrics, are presented in **Table 1**. For the age distribution, the average onset age in our study population was 41.6 ± 14.9 years old, and the vast majority of patients (19/21, 90.5%) were aged 20 to 60 years. The 20s and 40s were observed as two peak periods of adenoma-related symptoms appearing, and further illustrations of age distribution are illustrated in **Figure 2**. There was a higher risk of disease among men than women, and the male-to-female ratio was approximately 3:2 (13:8). A delayed diagnosis was observable in over half of the participants (12/21, 57.1%).

**Table 1:** Baseline characteristics of 21 GH/TSH cosecreting adenoma patients.

Items	Numerical value
Age of admission (y)	45.1 ± 13.7
Male (n, %)	13 (61.9%)
Age of symptom onset (y)	41.6 ± 14.9
Delay diagnosis (n, %)	12 (57.1%)
BMI (kg/m^2^)	25.4 ± 2.7
Systolic blood pressure (mmHg)	127.1 ± 13.8
Diastolic blood pressure (mmHg)	76.4 ± 10.0
Pulse rate (bpm)	84.0 ± 12.6

BMI, body mass index.

### 3.2 Clinical manifestations, hormonal, imaging and pathological characteristics of participants with GH/TSH coexisting tumors

Different symptoms of patients traced to their first visit to medical institutions among 21 mixed GH/TSH adenomas. The first symptom of 10 cases (47.6%) was related to thyrotoxicosis, whereas complaints of 6 patients (28.6%) were related to oversecretion of GH at the beginning of the disease course. In addition, 3 patients (14.3%) and 2 patients (9.5%) were admitted to hospitals first for mass effects (including headache and impaired visual function) and hypogonadotropic hypogonadism, respectively. During the whole follow-up of cases, more symptoms or signs tended to present in the same patients, and the number and percentage of total detailed clinical manifestations are presented in **Table 2**.

**Table 2:** Various clinical manifestations of GH/TSH cosecreting adenoma patients.

	No.	% of total
Manifestations related to GH excess		
Acral enlargement	9	42.6
Facial dysmorphia	7	33.3
Hyperhidrosis	13	61.9
Snoring or sleep apnea	9	42.6
Arthralgia	2	9.5
Accelerated growth in height	1	4.8
Weight gain	1	4.8
Manifestations related to TSH excess		
Palpitations	10	47.6
Tremor	4	19.0
Heat intolerance	3	14.3
Irritability	4	19.0
Increased appetite	6	28.6
Emaciation	6	28.6
Hyperdefecation	3	14.3
Manifestations related to tumor mass effects		
Headache	5	23.8
Visual impairment	7	33.3
Manifestations of hypopituitarism		
Sexual dysfunctions in the male	3	14.3
Menstrual disturbances in the female	1	4.8

GH, growth hormone; TSH, thyroid stimulating hormone.

The median GH, IGF-1/ULN, TSH, and thyroid hormone levels at baseline are listed in **Table 3**. Complete OGTT data were available, and the median nadir-GH was 1.8 ng/ml. Median PRL was 9.4 ng/ml, and mild elevation of PRL was found in 2 patients (43.8 ng/ml and 49.7 ng/ml) with negative staining of PRL in PA tissues. The median GH suppression rate and TSH suppression rate in OCT were 79.1% and 94.7%, respectively. All 21 mixed PAs were macroadenomas, and giant adenomas accounted for 23.8% (5/21). Among 21 mixed GH/TSH PAs, bone destruction in the sellar floor was observed in 33.3% of patients (7/21), and cavernous sinus invasion was observed in 57.1% of patients (12/21). In 38.1% (8/21) of cases, the Knosp grade was 3-4. Through thyroid ultrasonography, thyroid nodules (10/21, 47.6%) and diffuse goiter (9/21, 42.9%) were common. One patient was diagnosed with papillary thyroid carcinoma and treated by thyroidectomy. Among 19 patients who underwent transsphenoidal pituitary adenomectomy, 9 (42.6%) PAs presented with a tough texture, and 10 tumors (52.6%) were completely resected in the first operation. Through pathological analysis of PA tissues, 15.7% (3/19) of PA tissues presented a Ki-67 index >3. Because one of the patients refused the immunohistochemistry (IHC) for financial reasons, clinicopathological results of 18 cases were available. All these 18 PAs showed GH-positive staining, and 1/3 (6/18) of PAs showed TSH-positive staining.

**Table 3:** Hormonal, imaging and pathological characteristics of patients with mixed GH/TSH adenoma.

Items	Numerical value
Laboratory examinations at baseline	
GH (ng/ml)	4.0 [1.6, 9.1]
IGF-1/ULN (ng/ml)	1.9 [1.2, 2.9]
Nadir-GH in OGTT (ng/ml) ^#^ *(N = 12)*	1.8 [0.7, 5.6]
TSH (μIU/ml)	3.4 [2.1, 6.8]
FT3 (pg/ml)	6.7 [5.0, 9.2]
FT4 (ng/dl)	2.6 [2.2, 3.6]
TT3(ng/ml)	2.3 [1.6, 2.9]
TT4(ug/dl)	14.2 [13.0, 19.3]
PRL(ng/ml)	9.4 [6.7, 14.8]
GH suppression rates in OCT (%)^#^ *(N = 8)*	79.1 [68.8, 82.0]
TSH suppression rates in OCT (%)^#^ *(N = 12)*	94.7 [88.2, 97.0]
MRI of pituitary adenoma	
Maximum diameter of tumor (mm)	24.0 [15.0, 36.0]
Macroadenoma (n, %)	21 (100%)
Giant adenoma (n, %)	5 (23.8%)
Optic nerve compression (n, %)	11 (52.4%)
Bone destruction in sellar floor (n, %)	7 (33.3%)
Cavernous sinus invasion (n, %)	12 (57.1%)
Knosp grade (n, %)	
Grade 1-2	13 (61.9%)
Grade 3-4	8 (38.1%)
Thyroid ultrasonography	
Thyroid nodules (n, %)	10 (47.6%)
Diffuse lesion (n, %)	9 (42.9%)
Papillary thyroid carcinoma (n, %)	1 (4.8%)
Intraoperative findings^#^ *(N = 19)*	
Complete resection of tumor (n, %)	10 (52.6%)
Tough texture of tumor (n, %)	9 (42.6%)
Pathology	
Ki-67 index^#^ *(N = 19)*	
0-1 (n, %)	14 (73.7%)
2-3 (n, %)	2 (10.5%)
>3 (n, %)	3 (15.8%)
GH positive staining (n, %)^#^ *(N =18)*	18 (100.0%)
TSH positive staining (n, %)^#^ *(N =18)*	6 (33.3%)

GH, growth hormone; IGF-1, insulin-like growth factor 1; ULN, upper limit of normal; OGTT, oral glucose tolerance test; TSH, thyroid stimulating hormone; FT3, free triiodothyronine; FT4, free thyroxine; TT3, total triiodothyronine; TT4, total thyroxine; PRL, prolactin; OCT, octreotide test; MRI, magnetic resonance imaging.

^#^: There was unavailable data in partial patients and the actual totality (N) was listed subsequently.

### 3.3 Treatment patterns and outcomes of patients

The treatment patterns are shown in **Table 4**. Surgery was considered as the first-line therapy among most participants (19/21, 90.5%), and 66.7% of patients received injections of somatostatin analogs (SSAs) before the operation. Four patients (19.0%) received comprehensive treatment, including SSAs, surgery and radiotherapy. Only two patients refused surgery and accepted SSAs as the only therapy. During the follow-up of no less than 24 months, 23.8% of patients (5/21) achieved long-term remission of the GH/IGF-1 axis (≥12 months before the last visit), and 38.1% of patients (8/21) achieved short-term remission (3-12 months) before their latest follow-up. Persistent nonremission of the GH/IGF-1 axis or relapse of disease was observed in 38.1% (8/21) of subjects. Since mixed PA secretes both GH and TSH, the TSH axis was also reexamined in management. At the last visit, complete remission (both GH/IGF-1 and TSH) presented in one-third of patients, partial remission (GH/IGF-1 axis or TSH axis) appeared in one-third of cases, whereas nonremission (neither GH/IGF-1 axis nor TSH axis) or relapse occurred in the other one-third of participants. The Ki-67 index in mixed GH/TSH PAs with different outcomes at the last visit was reviewed and summarized. We found the Ki-67 index of the patients who achieved complete or partial remission were all 1%, whereas the Ki-67 index of the cases who stayed in nonremission ranged from 2% to 8%, which was higher than the former (**Table S1**, **Figure S1**).

**Table 4:** Treatment patterns and follow-up of 21 GH/TSH coexisting PAs.

	No.	% of total
Treatment regimen		
Surgery alone	5	23.8
SSAs alone	2	9.5
Preoperative SSAs and surgery	9	42.9
Surgery combined with preoperative and postoperative SSAs	1	4.8
SSAs, surgery and radiotherapy	4	19.0
Outcome of GH/IGF-1 axis during follow-up		
Long-term remission	5	23.8
Short-term and subsequently persistent remission	8	38.1
Persistent nonremission or relapse	8	38.1
Outcome of TSH axis during follow-up		
Long-term remission	11	52.4
Short-term and subsequently persistent remission	2	9.5
Persistent nonremission or relapse	8	38.1
Outcome of last visit		
Complete remission	7	33.3
Remission of GH/IGF-1	4	19.0
Remission of TSH	3	14.3
Nonremission	7	33.3

GH, growth hormone; IGF-1, insulin-like growth factor 1; TSH, thyroid stimulating hormone; SSAs, somatostatin analogues.

**Figure 3:**
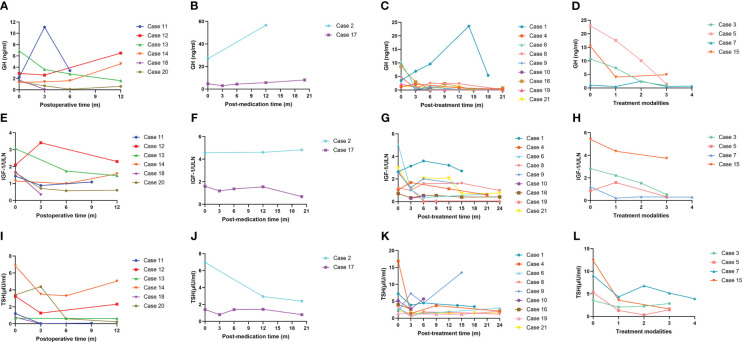
GH, IGF-1/ULN and TSH levels during treatment. GH, growth hormone; IGF-1, insulin-like growth factor 1; ULN, upper limit of normal; TSH, thyroid stimulating hormone. **(A)**, **(E)** and **(I)**: Changes in GH, IGF-1/ULN and TSH levels among patients who received only surgical treatment. **(B)**, **(F)** and **(J)**: Changes in GH, IGF-1/ULN and TSH levels among patients who received only SSA treatment. **(C)**, **(G)** and **(K)**: Changes in GH, IGF-1/ULN and TSH levels among patients who received surgery combined with preoperative and postoperative SSAs. **(D)**, **(H)** and **(L)**: Changes in GH, IGF-1/ULN and TSH levels among patients who accepted three therapies, including SSAs, surgery and radiotherapy.

**Figure 4:**
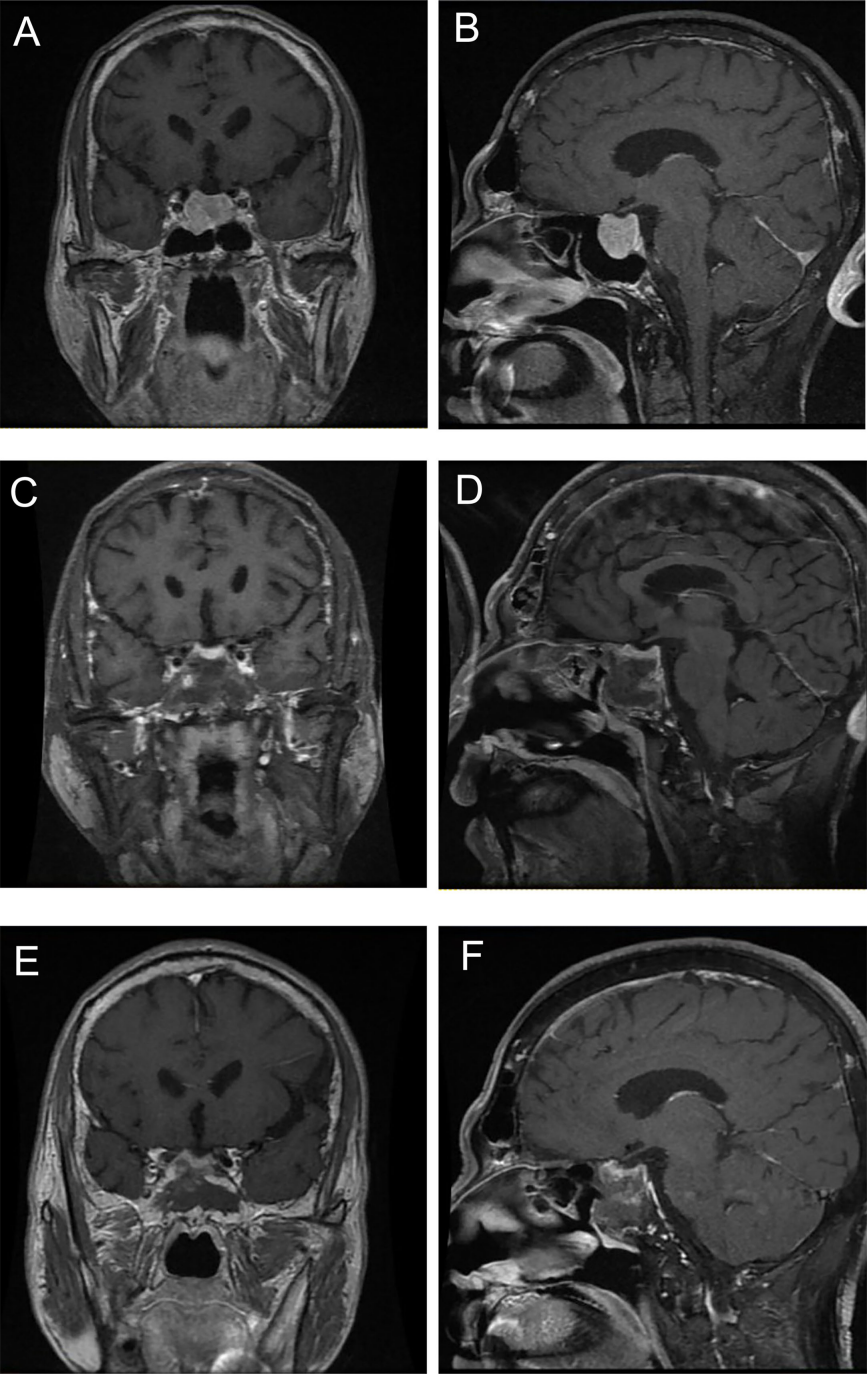
The MRI images of a GH/TSH cosecreting pituitary adenoma at different treatment stages. **(A)** and **(B)** were taken before surgery, **(C)** and **(D)** were taken at third month after surgery, **(E)** and **(F)** were taken at fifth year after radiotherapy.

Changes in the circulating GH, IGF-1/ULN and TSH during treatment are illustrated in **Figure 3**, and the levels of FT4 and FT3 during treatment are displayed in **Figure S2**. Among patients treated with surgery alone (n = 6), levels of GH, IGF-1/ULN, TSH, FT4 and FT3 were dramatically decreased three months after surgery, except for GH of Case 11, IGF-1/ULN of Case 12 and TSH of Case 20. However, the levels of the first three hormones in Case 14 subsequently rose again. The concentration of GH, TSH, FT4 and FT3 in Case 12 also increased at the 12th month after the operation. Only two patients accepted SSAs as the only option. Both Case 2 and Case 17 received regular injections of long-term octreotide (20 mg every 4 weeks). The hormones of Case 17 were slightly higher than the upper limits of normal at baseline and remained relatively stable during treatment. Nevertheless, five hormones of Case 2 were significantly higher at baseline and stayed relatively high throughout the follow-up. As is illustrated in **Figures 3C–G** and **Figures S2C–G**, surgery combined with preoperative SSAs contributed to the relative stability of hormones. These 9 patients were administered intramuscular SSAs three times before the operation, during which five hormones were clearly reduced in most cases. Case 9 received injection of long-acting octreotide (20 mg, only once) in the 4 weeks before surgery. Case 4, 6, 8, 16, and 19 received preoperative injection of long-acting octreotide (20 mg every 4 weeks) for 3 times. Case 10 received preoperative injection of long-acting octreotide (20 mg every 4 weeks) for a total of 4 times, Case 21 and Case 1 received preoperative injection of long-acting octreotide (20 mg every 4 weeks) for a total of 11 times and 14 times, respectively. Sustained attention was given to the hormone levels with superimposition of multiple treatment modalities in the remaining 4 cases treated with surgery, SSAs and radiotherapy. The order of therapy modalities was mostly preoperative SSAs first (Treatment modality 1), pituitary adenomectomy next (Treatment modality 2), followed by radiotherapy (Treatment modality 3). Case 3 and Case 7 received three injections of long-acting octreotide (20mg every 4 weeks) treatment before surgery, and Case 5 received six injections of long-acting lanreotide (40 mg every 4 weeks) and two injections of long-acting octreotide (20 mg every 4 weeks) treatment first, and then the above three patients received surgery and 25 times of radiotherapy. The order of treatment modalities was slightly different in Case 15. Case 15 underwent surgery first, followed by 3 injections of long-term octreotide (20 mg every 4 weeks) treatment and radiotherapy. As shown in **Figures 3D–L** and **Figures S2D–H**, the rising trend of five hormones before surgery (Treatment modality 1 to 2) was visible. Then, they underwent surgery, and their hormones continued to fall and subsequently remained steady (Treatment modalities 2 to 3). After radiotherapy at the latest visit (Treatment modality 3), oversecretion of hormones among them was ideally controlled. Case 15, a 55-year-old female, received surgery initially and accepted SSA injections and radiotherapy sequentially; nevertheless, biological remission had not been achieved during integrative therapy for nearly 5 years. The MRI images of Case 5, who received comprehensive treatment combined with SSAs, surgery and radiotherapy, is shown in **Figure 4**. After three injections of long-acting lanreotide (40mg every 14 days), this 49-year-old male accepted endoscopic transsphenoidal pituitary adenomectomy and the initial macroadenoma (23 mm×22 mm×21mm) became invisible on MRI **(Figures 4A, D**). At the third month after surgery, he accepted radiotherapy and the pituitary region remained stable at the fifth year after the radiotherapy (**Figures 4E, F**).

### 3.4 Comparative study of GH/TSH cosecreting and GH monosecreting PAs

We retrospectively reviewed 42 age- and sex-matched GH monosecreting PA cases and compared several aspects of their baseline characteristics with those of the GH/TSH cosecreting group. As shown in **Table 5**, age and sex were comparable between the two groups. Baseline levels of GH and IGF-1/ULN were higher in GH monosecreting PAs than in GH/TSH cosecreting PAs (GH: 13.7 [4.8, 25.4] ng/ml *vs*. 4.0 [1.6, 9.1] ng/ml, P = 0.002; IGF-1/ULN: 3.1 [2.5, 3.9] *vs*. 1.9 [1.2, 2.9], P = 0.003). According to pituitary MRI, the average maximum diameter of the tumor was higher in the GH/TSH cosecreting PA group than in the GH monosecreting PA group (24.0 [15.0, 36.0] mm *vs*. 14.7 [10.8, 23.0] mm, P = 0.005). There was no difference in the incidence of bone destruction in the sellar floor (P = 0.422), whereas cavernous sinus invasion was more significantly observable in the GH/TSH cosecreting PA group than in the GH monosecreting PA group (57.1% *vs*. 23.8%, P = 0.009). A trend of a higher presence of Knosp grade 3-4 was found in GH monosecreting PA patients (P = 0.052). GH/TSH cosecreting PA subjects showed a higher prevalence of arrhythmia (especially atrial fibrillation), heart enlargement and osteopenia/osteoporosis; however, no significantly different occurrence of hypertension, abnormal glucose metabolism and hyperlipidemia was observed between two groups.

**Table 5:** Comparison of characteristics between GH/TSH cosecreting adenoma and GH monosecreting adenoma groups.

Characteristics	GH/TSH cosecreting PA group (n = 21)	GH monosecreting PA group (n = 42)	P value
Age of admission (y)	48.3 ± 13.3	45.9 ± 9.4	0.411
Male (n, %)	13 (61.9%)	26 (61.9%)	1.000
GH (ng/ml)	4.0 [1.6, 9.1]	13.7 [4.8, 25.4]	0.002^*^
IGF-1/ULN	1.9 [1.2, 2.9]	3.1 [2.5, 3.9]	0.003^*^
Maximum diameter of tumor (mm)	24.0 [15.0, 36.0]	14.7 [10.8, 23.0]	0.005^*^
Bone destruction in sellar floor (n, %)	7 (33.3%)	10 (23.8%)	0.422
Cavernous sinus invasion (n, %)	12 (57.1%)	10 (23.8%)	0.009^*^
Knosp grade 3-4 (n, %)	8 (38.1%)	6 (14.3%)	0.052
Complications			
Arrhythmia (n, %)	6 (28.6%)	1 (2.4%)	0.004^*^
Heart enlargement (n, %)	7 (33.3%)	2 (4.8%)	0.005^*^
Osteopenia/osteoporosis (n, %)	7 (33.3%)	1 (2.4%)	0.001^*^
Hypertension (n, %)	5 (23.8%)	14 (33.3%)	0.437
Abnormal glucose metabolism (n, %)	11 (52.4%)	15 (35.7)	0.205
Hyperlipidemia (n, %)	1 (4.8%)	3 (7.1%)	0.999
Surgical technique			0.332
Microscope transsphenoidal surgery	13 (68.4%)^#^	34 (81.0%)	
Endoscopic transsphenoidal surgery	6 (31.6%)^#^	8 (19.0%)	
Complete resection of tumor	10 (52.6%)	37 (88.1%)	0.006^*^
Integrative treatment (n, %)	4 (19%)	6 (14.3%)	0.719
Outcome of follow-up			
Long-term remission (n, %)	6 (28.6%)	30 (71.4%)	<0.001^*^
Short-term remission (n, %)	2 (9.5%)	0 (0%)	0.256
Persistent non-remission or relapse (n, %)	13 (61.9%)	12 (28.6%)	0.019^*^

GH, growth hormone; TSH, thyroid stimulating hormone; IGF-1, insulin-like growth factor 1; ULN, upper limit of normal.

P values indicate statistical significance. ^*^P <0.05.

^#^: Nineteen patients with mixed GH/TSH PA underwent surgery, and the remaining two patients accepted SSAs as treatment.

There are 19 patients in GH/TSH cosecreting PA group and 42 patients in GH monosecreting PA group who underwent surgery (**Table 5**). Either microscope transsphenoidal surgery or endoscopic transsphenoidal surgery was chosen in the included cases and no significant difference of surgical approach was observed between two groups (P = 0.332). In order to ascertain whether the tumor was completely resected or not, pituitary MRI within three months after surgery was carefully checked. The proportion of completely resected tumors is significantly lower in GH/TSH cosecreting PA group compared to that in GH monosecreting PA group (52.6% *vs*. 88.1%, P = 0.006). As for the outcome of GH/IGF-1 during the follow-up, long-term remission was defined as continuous biochemical remission of no less than 12 months before the last visit. The cases remained in biochemical remission for no less than 3 months but less than 12 months. The percentage of long-term remission cases was higher in the GH monosecreting PA group (71.4% *vs*. 28.6%, P <0.001), and persistent nonremission or relapse was more common in the GH/TSH cosecreting PA group (61.9% *vs*. 28.6%, P = 0.019).

## 4 Discussion

According to previous studies, approximately 16.0%**-**19.7% of TSHomas cosecret GH, whereas the percentage of mixed GH/TSH PAs in GH-secreting pituitary tumors was lower in previous reports (9, 10, 22). Based on the review of GHPA cases admitted to our pituitary center, GH/TSH cosecreting PAs account for only 1.4% of GHPAs, verifying the rarity of this bihormonal adenoma. Recent updates on the epidemiology revealed that the median age at diagnosis ranges from 40.5 to 47 years old, and most TSHoma patients were diagnosed between the ages of 40 and 60 (23, 24). In this study, most patients with mixed GH/TSH PAs were admitted to our hospital and correctly diagnosed between the ages of 31.4 and 58.8 years old. PA-related symptoms appeared before 40 years old in 47.5% (10/21) of GH/TSH coexisting PA patients (**Figure 2**), which was earlier than that among monohormonal PAs in the literature. This phenomenon implied that it is essential to comprehensively screen and regularly reexamine the pituitary hormones in young and middle-aged PA patients to clarify the presence or absence of bihormonal PAs.

Due to the lack of awareness of related symptoms and signs at the early stage of GHPA, it often takes a long time to be aware of the typical manifestations, including facial dysmorphia, acral enlargement, abnormal glucose metabolism, and various complications. The thyrotoxicosis caused by TSHomas is frequently lighter than primary hyperthyroidism. The correct diagnosis of TSHomas requires hormone detection, pituitary MRI and even genetic testing. The above two points may explain why delayed diagnosis existed in over half of the included cases. No significant sex differences have been reported in pure GHPA or TSHomas, whereas the incidence of mixed PAs was much higher in males in the present study (23, 24).

In the process of screening our patients, a GH/TSH cosecreting PA case diagnosed as multiple endocrine neoplasia type 1 (MEN1) caught our attention. Regrettably, she was excluded because a giant space-occupying lesion of the liver was found in the same time period, and this 58-year-old female died of hepatocellular carcinoma 5 months later. An investigation of her family history showed that her sister passed away for pancreatic cancer and hepatic cirrhosis years ago. A cohort study on PA in MEN1 patients showed that prolactinomas and NFPAs accounted for 45.5% and 36.1%, respectively (25). Thus, we found a peculiar MEN1 case with GH/TSH coexisting PA. Therefore, we suggest that clinical lineage whole exome sequencing should be advocated for patients with a family history of high-risk tumors, which is helpful in filling the gap in the genetic knowledge of mixed GH/TSH PAs.

Similar to previous studies, the major manifestations of GH/TSH coexisting PAs include acromegaly, hyperthyroidism with or without goiter, the mass effects of tumors and other axes involved (11, 13–15, 17, 21). Although the most common first symptom was hyperthyroidism, their thyroid antibodies were negative, and a series of symptoms frequently observed in thyroid disease did not occur in these patients, such as exophthalmos and mucous edema. Because of the obscure symptoms of inappropriate secretion of TSH and acromegaly, the ignorance and delayed diagnosis of disease often occurs. The major manifestations of the affected gonadal axis in our patients were central hypogonadism or reduced libido in the males and menstrual disturbance in the females, which was consistent with current reports (11, 14, 26–28). In view of the exclusion of mixed prolactinoma, the pituitary stalk effect might contribute to the slight elevation of PRL in two of our patients without any symptoms or signs of hyperprolactinemia. All the included GH/TSH cosecreting PAs were macroadenomas, and nearly one-quarter of the PAs were giant adenomas. The mixed PA group presented with a larger median maximum diameter and more cavernous sinus invasion than the age- and sex-matched GH monosecreting PA group, verifying the deductions from a few researchers that PIT-1 lineage plurihormonal PAs are frequently large, and they are also more invasive than mono-secreting PAs (12, 14, 29). The overexpression of PIT-1 is possibly related to cell proliferation in GHPAs, PRLomas and TSHomas, even though the pathophysiological mechanism has not been fully investigated (14, 30). Moreover, excessive GH and TSH might additively provide permissive conditions for the proliferation and growth of PA (31–33). Conversion to malignant tumor or metastatic spread did not appear in the participants. The levels of GH and IGF-1 seemed lower in the mixed PA group. Based on the knowledge that GH-secreting somatotrophs and TSH-secreting thyrotrophs are both derived from the process of differentiation in PIT-1-positive cells, Sanada et al. assumed that GH and TSH were possibly produced by the same cells in adenoma tissue (13). Thus, we speculate that these cells are immature with a lower degree of differentiation and less specialized production of GH than the adenoma cells in GHPAs, leading to lower GH and IGF-1 at baseline in the mixed PA group than in the GHPA controls.

Among 65 patients diagnosed with TSH-producing pituitary adenomas who were admitted to Peking Union Medical College Hospital during 2011 and 2020, 23 patients (35.4%) were immunostained negative for TSH, and they achieved thyroid hormone plunge after surgery and remission, suggesting that a considerable fraction of TSH-producing PAs might be negative TSH staining (34). The immunohistochemical staining of TSH in two-thirds of mixed PAs was negative, and there are multiple possible reasons. Firstly, the PIT-1-positive plurihormonal tumor has been divided into the immature PIT-1-lineage tumor and the mature plurihormonal PIT-1-lineage tumor in the 2022 WHO classification of pituitary tumors (2). These tumors, especially the immature PIT-1-lineage tumor, are often plurihormonal and do not show terminal differentiation based on morphology, immunoprofile, ultrastructure, and function (2). On account of the incomplete differentiation of immature PIT-1-lineage tumor, there are monomorphic tumor cells with focal/variable staining for no hormones, or one or more of GH, PRL, β-TSH, and/or α-subunit in the tumor (2). Even in the IHC of mature plurihormonal PIT-1-lineage tumor, GH is expressed predominantly, whereas other hormones are expressed variably (2). We speculated some of the included mixed GH/TSH pituitary adenomas were immature PIT-1-lineage tumor, which may explain some tumors were stained negative for TSH. Secondly, the abnormal and pathological biosynthesis, transport and secretion of TSH might contribute to the negative immunostaining. TSH is secreted in pituitary under the regulation of thyrotropin releasing hormone (TRH), somatostatin, dopamine and thyroid hormones, following a diurnal pattern with small bursts (35). However, TSH secretion is disorganized in tumor cells may be due to the altered functional properties within the TSH-producing PAs (36). The increased secretion of TSH may give rise to relatively deceased storage of TSH in cytoplasm. According to the study by Schroeder et al., the similar absence of GH in IHC was also observed in some cases of pituitary somatotroph tumors, demonstrating GH immunostaining may not be sufficient for the diagnosis of GHPAs (37). The researchers think that the GH was secreted quickly by these tumor cells after the synthesis, therefore, the cytoplasm level of GH is too low to be detected by IHC, leading to the negative staining (37). We supposed that the same principle applies to TSH-producing pituitary adenomas. Thirdly, it is possible that the relatively ubiquitous fibrosis of tumor obstructs the binding of antigen and antibody in IHC. In approximately 40% of the thyrotroph tumors, fibrotic characteristics are seen, which is different from other subtypes of pituitary adenomas, making it slightly firm (36, 38). The thyrotroph tumor becomes denser with the higher degree of fibrosis, and some tumors may even appear calcified. The antigen-binding capacity of antibody in IHC might slide into decline due to the fibrosis of tumor tissues. In previously reported cases from other centers, the negative staining of TSH turns positive after the application of proteinase K, which may be not sensitive enough to the antibodies selected for the detection of TSH or combined with certain unknown factors (39, 40).

Unlike other NETs, PitNETs are not stratified into grades based on their Ki-67 index. “Atypical adenoma” was defined by the WHO in 2004 as Ki-67 labeling index >3%, increased fission and positive p53 immunostaining, which were considered to behave aggressively. However, not all PAs that meet the criteria present an aggressive course, and even some non-aggressive PAs may meet the criteria. As a result, “atypical adenoma” was deleted in the 4th edition of the WHO classification in 2017. Therefore, the Ki-67 index has been a controversial marker for determining the aggressive pattern of PAs. In our study, the Ki-67 index was significantly higher in the mixed GH/TSH PAs without complete remission, suggesting the potential association between Ki-67 index the aggressive course of GH/TSH cosecreting PAs. The multi-center study investigating larger number of cases are warranted to verify this correlation.

Thyroid lesions emerged in one hundred percent of our cases diagnosed as mixed PA, including thyroid nodules, diffuse lesions and thyroid carcinoma. The occurrence of thyromegaly was higher in GHPAs and TSHomas than in controls in current studies (41–43). The possible mechanism was that IGF-1 plays an important role in increased cellular proliferation and decreased cellular apoptosis in the enlargement of multiple organs, including the thyroid (42, 44). The receptors of IGF-1 are specifically expressed in follicular cells of the thyroid (45). Furthermore, TSH may influence the biological effects of GH and IGF-I on the thyroid as well as other target organs or tissues (45). One patient was diagnosed with papillary thyroid carcinoma and underwent thyroid lobectomy. Mixed PAs complicated with thyroid cancer have rarely been reported and are challenging to treat because of the complex interactions between hormones (12, 18). Hence, clinicians should be vigilant to screen the thyroid and prevent misdiagnosis. Many studies have indicated that the prevalence rates of a series of cardiovascular diseases are elevated in acromegaly, including cardiomyopathy, arrhythmia and hypertension (46–49). There is a paucity of research on the cardiovascular complications of TSHomas, except for a few reports referring to the correlation between TSHomas and clinical or subclinical atrial fibrillation (50, 51). We speculate that the duplicated effects of GH and TSH gave rise to the higher incidence rate of arrhythmia and heart enlargement in the mixed PA group. This two-way interplay might have remarkable impacts on bone metabolism; therefore, the GH/TSH coexisting group seemed more susceptible to osteopenia/osteoporosis than the GH mono-secreting group in our research.

The new WHO classifications of PAs mention that plurihormonal PIT-1 lineage adenomas might be aggressive in terms of their size, growth rate, invasiveness and recurrence (2, 29). We found a similar phenomenon in PAs cosecreting GH and TSH in that they displayed refractory features with a lower rate of long-term remission and a higher incidence rate of persistent nonremission or relapse, implying a worse prognosis. According to the existing guidelines and consensus, the first-line treatment for GH and TSH monosecreting PAs is surgery, which may not completely cure mixed GH/TSH PAs on account of their fibrotic nature and invasiveness (9, 52, 53). Characteristically tough texture and more common cavernous sinus invasion displayed in bihormonal PAs restrained them from total resection in this study. According to previous studies, fibrotic characteristics are seen in approximately 40% of thyrotroph tumors, which is different from other subtypes of pituitary adenomas including somatotroph tumors (36, 38). Similarly, tough texture of tumor was found in 42.6% of bihormonal PAs in our study. Postoperative medications or adjuvant radiotherapy could be considered among these cases. As PIT-1 lineage PAs, both GH and TSH mono-secreting PAs express somatostatin receptors (SSTRs), especially SSTR2 and SSTR5, which can be inhibited by somatostatin analogs (SSAs) (54–56). Our study demonstrated that octreotide, a widely applied first-generation SSA, could suppress octreotide baseline GH and TSH levels by 79.1% and 94.7%, respectively. Additionally, circulating GH, IGF-1 and TSH apparently declined in the first three months of SSA application. In this sense, the choice of SSA injection as initial treatment is conducive to rapid control of the oversecreted hormones and efficacy of subsequent surgery. Indeed, it seemed that comprehensive therapeutic regimens comprised of two or three modalities were preferred in mixed GH/TSH PA cases in this research. With the increase and accumulation of treatment modalities, the concentrations of GH, IGF-1 and TSH declined gradually and eventually stabilized, suggesting that the combination of multiple modalities should be advocated in refractory mixed PAs. Radiotherapy might be conducive to the long-term remission and stability of disease.

Once the diagnosis of GH/TSH cosecreting PA is established, a vigorous treatment strategy should be made under the full discussion of a pituitary multidisciplinary team (MDT) including endocrinologists, neurosurgeons, radiologists, neuroradiologists, pathologists and so on. Early detection and prompt diagnosis are crucial for patients with GH/TSH coexisting PAs, and regular consultation and careful follow-up of these cases are needed. In summary, clinicians should bear in mind the possibility of bihormonal PAs at the same time, especially in patients with GHPAs or TSHomas.

Because of the extreme rarity of GH/TSH cosecreting PAs, the majority of these special bihormonal PIT-1 adenomas have been described only in case reports. To our knowledge, this study has comprehensively reviewed the largest number of cases diagnosed with mixed GH/TSH PAs in terms of their clinical manifestations, hormone levels, imaging features, therapy modalities, therapeutic responses and follow-up outcomes. In addition, a comparative analysis between GHPA coexisting with GH monosecreting PAs was performed for the first time. We hold the opinion that this study may provide enlightenment to many clinicians in the awareness and understanding of bihormonal PAs. Due to the extreme rarity of GH/TSH cosecreting PA, the sample size was inevitably limited. Besides, underlying bias in a single-center study cannot be excluded, and multicenter research is warranted. In addition, the process of follow-up was affected by the outbreak of COVID-19. Thus, longer studies of ongoing clinical biochemical and radiological follow-up among these patients are needed.

## Conclusion

Compared to age- and sex-matched GH mono-secreting PAs, coexisting GH/TSH PAs appeared larger, more invasive and refractory with a worse prognosis. The diagnosis and treatment of GH/TSH cosecreting PAs has been a great challenge. A multidisciplinary treatment strategy of surgery combined with medications or radiotherapy was more effective than surgery alone in managing this disease.

## Data availability statement

The raw data supporting the conclusions of this article will be made available by the authors, without undue reservation.

## Author contributions

All authors of the study contributed significantly. Detailed author contributions: conceptualization: NY and HZ; methodology: NY, LD, SY, and HZ; investigation: NY, FH, SY, JL, MC, and LD; software: NY; data curation: NY, FH, and MC; writing-original draft preparation: NY; resources: YY, KD, FF, XL, XM, LD, and HZ; supervision: HZ. All authors have read and approved the final manuscript.

## Supplementary material

Supplementary Figure 1 The Ki-67 index of mixed GH/TSH PAs.

Supplementary Figure 2 FT4 and FT3 levels during treatment. FT4, free thyroxine; FT3, free triiodothyronine. **(A)** and **(E)**: Changes in FT4 and FT3 levels among patients who received only surgical treatment. **(B)** and **(F)**: Changes in FT4 and FT3 levels among patients who received only SSA treatment. **(C)** and **(G)**: Changes in FT4 and FT3 levels among patients who received surgery combined with preoperative and postoperative SSAs. **(D)** and **(H)**: Changes in FT4 and FT3 levels among patients who accepted three therapies, including SSAs, surgery and radiotherapy.
