# Supplementary figures and images for: Clinical features and therapeutic outcomes of GH/TSH cosecreting pituitary adenomas: experience of a single pituitary center
Source: Front Endocrinol (Lausanne). 2023 May 30;14:1197244. doi: 10.3389/fendo.2023.1197244 (PMC10265640; doi:10.3389/fendo.2023.1197244)

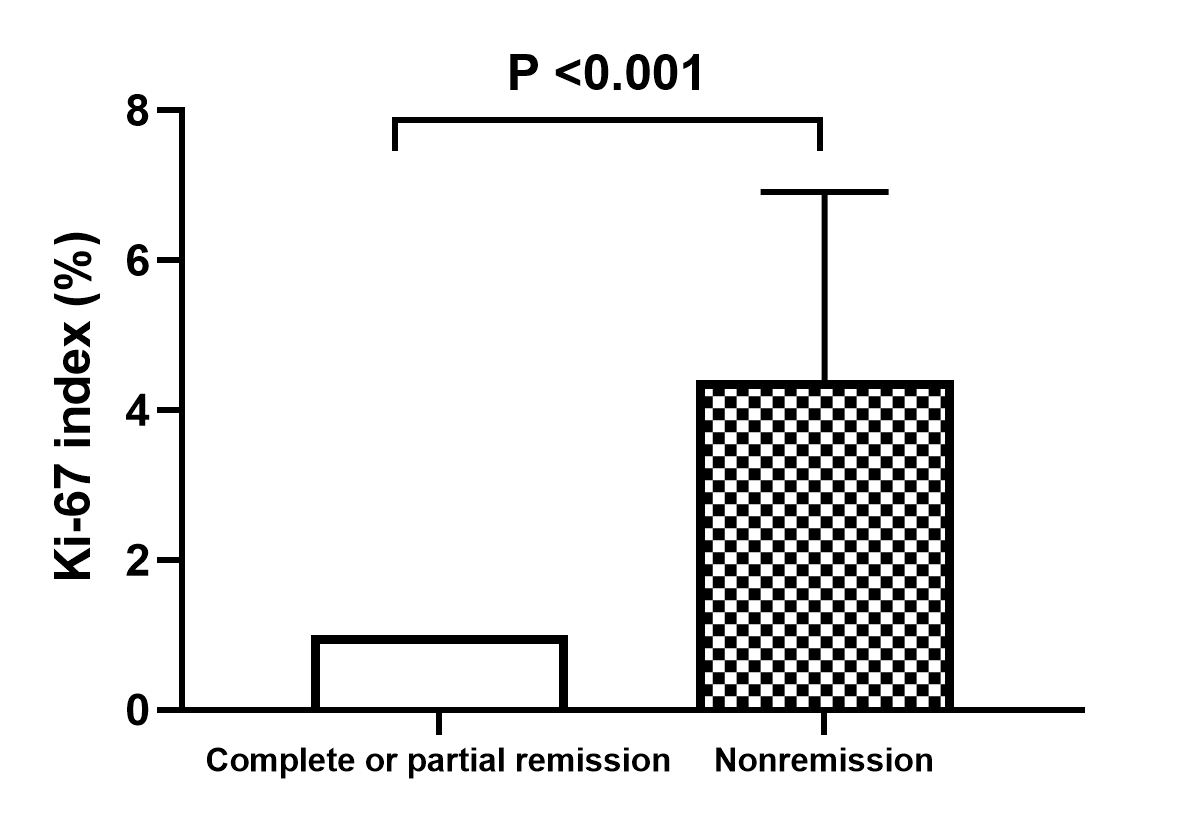

Supplement: Supplementary Figure 1 — The Ki-67 index of mixed GH/TSH PAs. [file Image_1.tif]

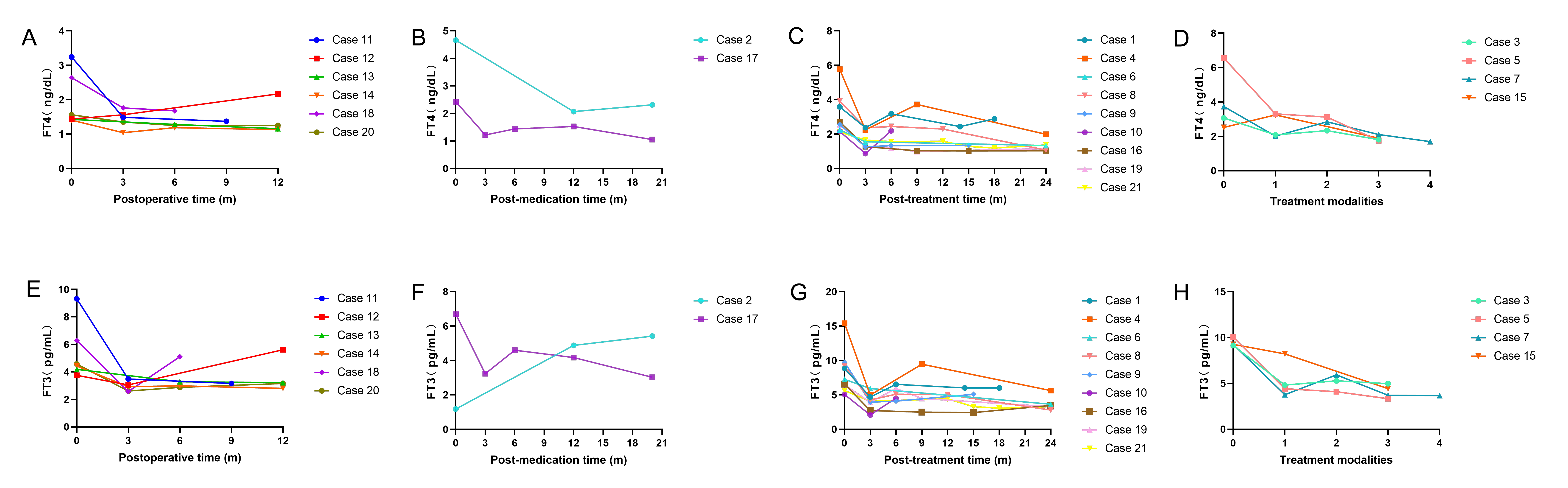

Supplement: Supplementary Figure 2 — FT4 and FT3 levels during treatment. FT4, free thyroxine; FT3, free triiodothyronine. (A) and (E): Changes in FT4 and FT3 levels among patients who received only surgical treatment. (B) and (F): Changes in FT4 and FT3 levels among patients who received only SSA treatment. (C) and (G): Changes in FT4 and FT3 levels among patients who received surgery combined with preoperative and postoperative SSAs. (D) and (H): Changes in FT4 and FT3 levels among patients who accepted three therapies, including SSAs, surgery and radiotherapy. [file Image_2.tif]
