# Supplementary material for: Clinical features and therapeutic outcomes of GH/TSH cosecreting pituitary adenomas: experience of a single pituitary center
Source: Front Endocrinol (Lausanne). 2023 May 30;14:1197244. doi: 10.3389/fendo.2023.1197244 (PMC10265640; doi:10.3389/fendo.2023.1197244)
Supplement: Supplementary file 3 [file Table_1.docx]

**Table S1. The Ki-67 index of included mixed GH/TSH PAs.**

| **Outcome of last visit** | **Ki-67 index (%)** | **P value** |
| --- | --- | --- |
| Complete or partial remission (n = 14) | 1 [1, 1] | ＜0.001^*^ |
| Nonremission (n = 5) | 5 [2, 5] |  |
